# Soluble Interleukin-6 Receptor Induces Motor Stereotypies and Co-Localizes with Gp130 in Regions Linked to Cortico-Striato-Thalamo-Cortical Circuits

**DOI:** 10.1371/journal.pone.0041623

**Published:** 2012-07-23

**Authors:** Ankur Patel, Youhua Zhu, Eldo V. Kuzhikandathil, William A. Banks, Allan Siegel, Steven S. Zalcman

**Affiliations:** 1 Department of Neurology and Neurosciences, New Jersey Medical School, University of Medicine and Dentistry of New Jersey, Newark, New Jersey, United States of America; 2 Department of Psychiatry, New Jersey Medical School, University of Medicine and Dentistry of New Jersey, Newark, New Jersey, United States of America; 3 Department of Pharmacology and Physiology, New Jersey Medical School, University of Medicine and Dentistry of New Jersey, Newark, New Jersey, United States of America; 4 Division of Gerontology and Geriatric Medicine, Department of Medicine, Geriatrics Research Education and Clinical Center, Veterans Affairs Puget Sound Health Care System, University of Washington, Seattle, Washington, United States of America; 5 Department of Neurology and Neurosciences, New Jersey Medical School, University of Medicine and Dentistry of New Jersey, Newark, New Jersey, United States of America; University of Jaén, Spain

## Abstract

Soluble cytokine receptors are normal constituents of body fluids that regulate peripheral cytokine and lymphoid activity and whose levels are increased in states of immune activation. Soluble interleukin-6 receptor (sIL-6R) levels positively correlate with disease progression in some autoimmune conditions and psychiatric disorders. Particularly strong links between levels of sIL-6R and the severity of psychotic symptoms occur in schizophrenia, raising the possibility that sIL-6R is involved in this disease. However, there is no evidence that peripheral sIL-6R induces relevant behavioral disturbances. We showed that single subcutaneous injections of sIL-6R (0–1 µg), stimulated novelty stress-induced exploratory motor behaviors in male Balb/c mice within 20–40-min of injection. A progressive increase in vertical stereotypies was observed 40–80 min post injection, persisting for the remainder of the test session. Paralleling these stimulant-like effects, sIL-6R pre-treatment significantly enhanced stereotypy scores following challenge with GBR 12909. We found that peripherally administered sIL-6R crossed the blood-brain barrier, localizing in brain regions associated with cortico-striatal-thalamo-cortical (CSTC) circuits, which are putative neuroanatomical substrates of disorders associated with repetitive stereotypies. Peripherally administered sIL-6R co-localized with gp130, a transmembrane protein involved in IL-6 trans-signaling, in the nucleus accumbens, caudate-putamen, motor and infralimbic cortices, and thalamic nuclei, but not with gp130 in the ventral tegmental area, substantia nigra, or sensorimotor cortex,. The results suggest that peripheral sIL-6R can act as a neuroimmune messenger, crossing the blood brain barrier (BBB) to selectively target CSTC circuits rich in IL-6 trans-signaling protein, and inducing repetitive stereotypies. As such sIL-6R may represent a novel therapeutic agent for relevant psychiatric disorders.

## Introduction

Soluble cytokine receptors (SCR) lack the extracellular portion of their membrane counterparts and can act as circulating binding proteins. SCRs are normal constituents of body fluids that regulate their target cytokines in antagonistic and agonistic ways [1]–[3]. The soluble receptor for IL-6 (sIL-6R) has a greater range of agonistic effects than most SCRs. For example, in addition to extending the half-life in blood of IL-6 (and other cytokines of IL-6 family), the sIL-6R/IL-6 complex interacts with Glycoprotein 130 (gp130), forming a multimeric signaling complex that activates downstream signal transduction pathways. It is also important to note that regulatory effects of sIL-6R/IL-6 extend to non-immune cells, including sympathetic and sensory neurons from neonatal superior cervical ganglia and embryonic dorsal root ganglia [4], [5], and Schwann cell progenitors [6].

Pronounced increases in sIL-6R is evident in certain disease states linked to immune activation. For example, serum levels of sIL-6R are increased in autoimmune disorders, which notably include rheumatoid arthritis and juvenile rheumatoid arthritis [7]–[9]. There are positive relations between sIL-6R levels and disease progression for rheumatoid arthritis [10] and multiple myeloma [11], [12]. Several studies suggest that chronic immune activation subsequent to an infectious process or autoimmune condition might contribute to the etiology of psychiatric disorders such as depression and schizophrenia [13]. Increased levels of soluble cytokine receptors, which reflect an immune activation, are evident in these disorders. In particular, there is a strong correlation between levels of sIL-6R and severity of illness in schizophrenic patients [14]; raising the possibility that sIL-6R can act as an etiological factor. Support for this idea was provided by Muller and colleagues who showed that the levels of sIL-6R in serum and cerebrospinal correlate with symptoms in schizophrenic patients (paranoid-hallucinatory syndrome) [15]. Furthermore, it was shown that treatment with antipsychotic drugs decreased sIL-6R levels [16], [17]. Based on these results, it was suggested that sIL-6R might play a role in the expression of positive symptoms in schizophrenics [15]. However, to date, no studies have directly evaluated the role of peripheral sIL-6R in the behavioral disturbances exhibited by psychiatric patients.

**Figure 1:**
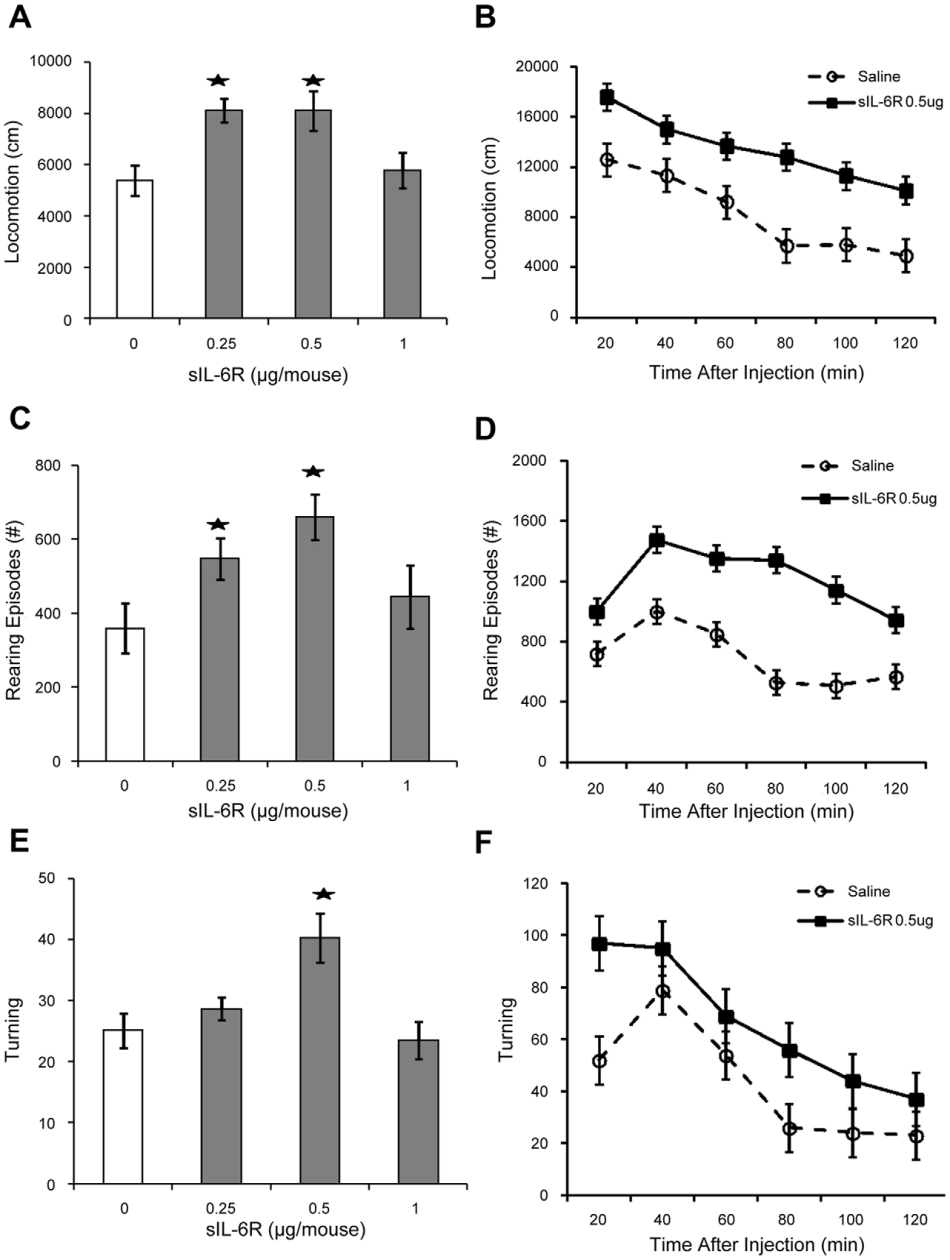
Behavioral effects induced by single injection of sIL-6Rα sIL-6R-induced behavioral changes. Mean (± S.E.M.) activity scores for; (A) Locomotion, (B) Locomotion over 20-min intervals, (C) Rearing, (D) Rearing episodes over 20-min intervals, (E) Turning and (F) Turning over 20-min intervals following single injections of sIL-6R (0, 0.25, 0.5 and 1 µg/mouse, sc). *p =  <0.05.

**Figure 2:**
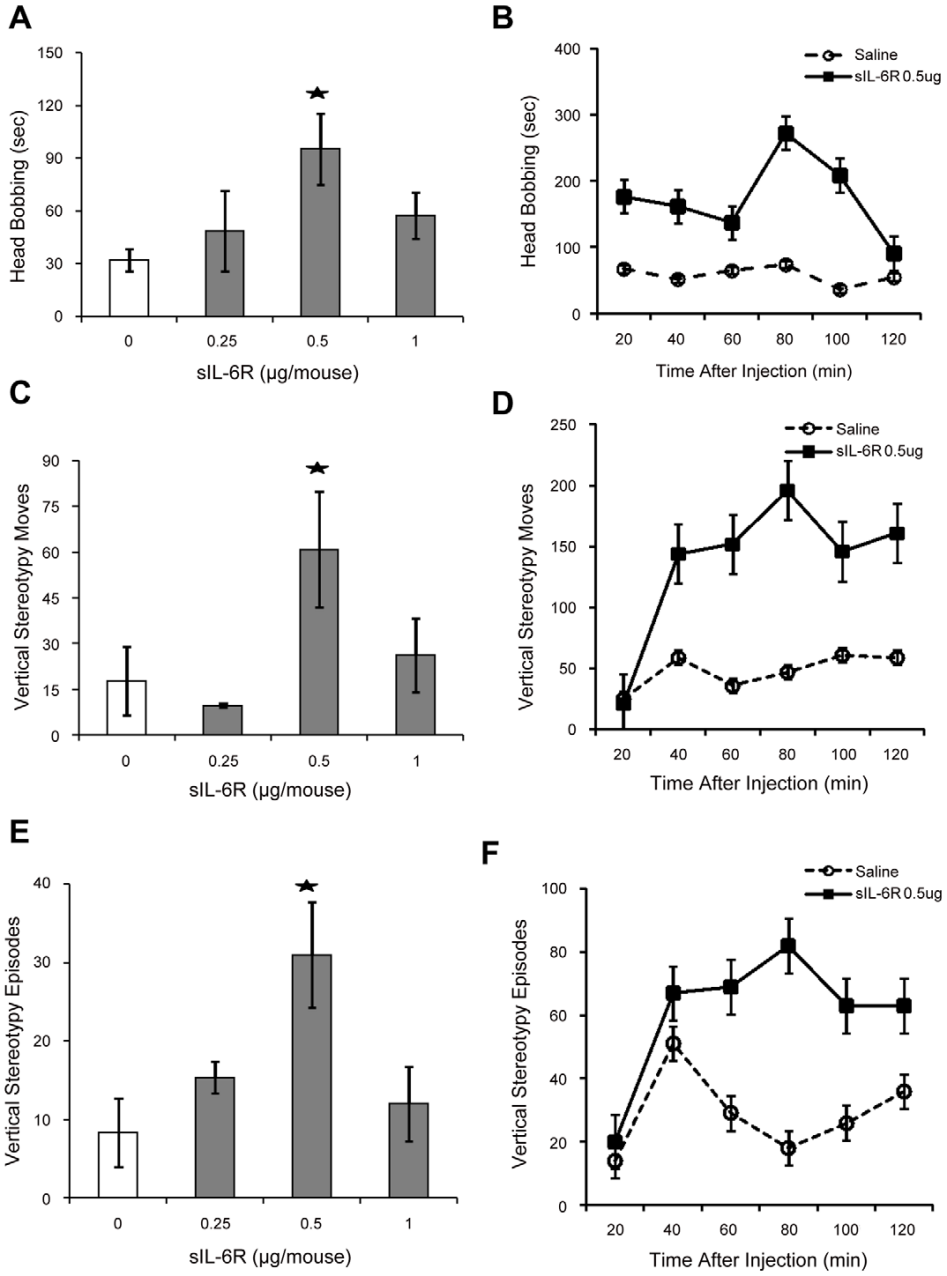
Stereotypic Behaviors induced by single injection of sIL-6Rα sIL-6R-induced repetitive stereotypic behavior. Mean (± S.E.M.) activity scores for; (A) Head bobbing, (B) Head bobbing over 20-min intervals, (C) Vertical Stereotypic Moves, (D) Vertical Stereotypic Moves over 20-min intervals, (E) Vertical Stereotypic Episodes and (F) Vertical Stereotypic Episodes over 20-min intervals following single injections of sIL-6R (0, 0.25, 0.5 and 1 µg/mouse, sc). *p =  <0.05.

**Figure 3:**
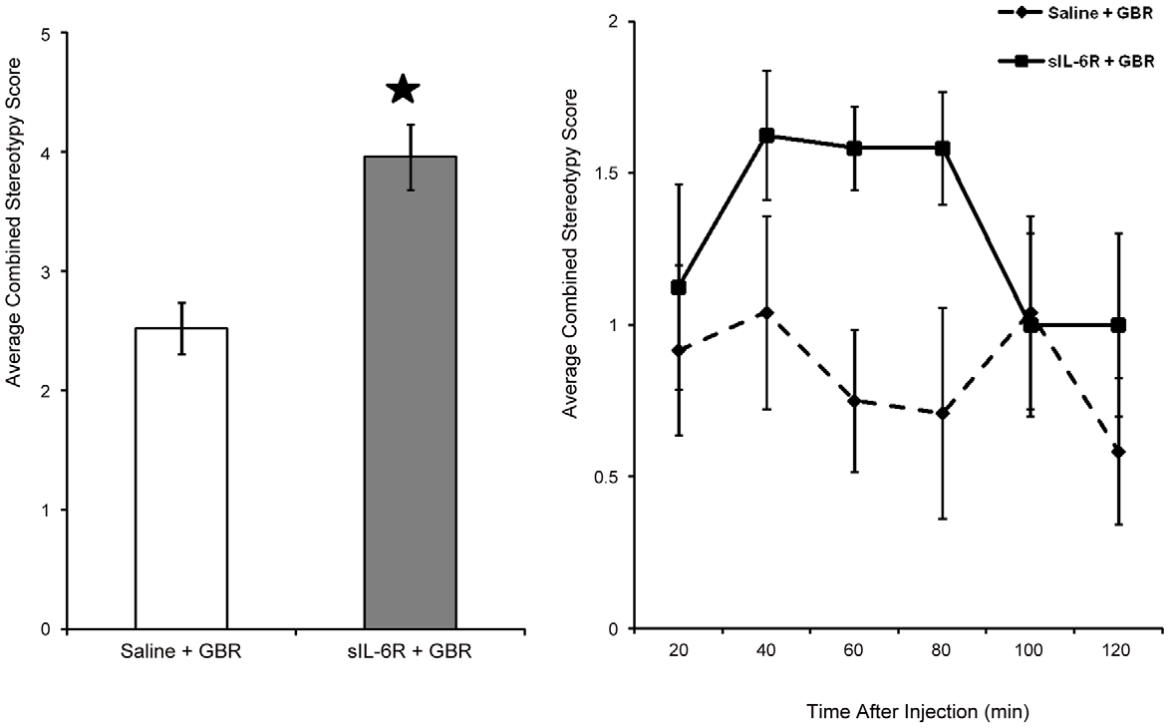
Effect of Saline or sIL-6Rα pretreatment on GBR induced behaviors. Stereotypy Score of effect of GBR on sIL-6R treated mice. Mean (± S.E.M.) activity for combined stereotypic score (A) and combined stereotypic score over 20-min intervals (B) following single injection of GBR (5 mg/kg) in saline and IL-6Rα (0.5 µg/mouse) treated mice. Combined stereotypic score includes average of repetitiveness, intensity and frequency of individual stereotypic behavior expressed by mice. *p =  <0.05.

**Figure 4:**
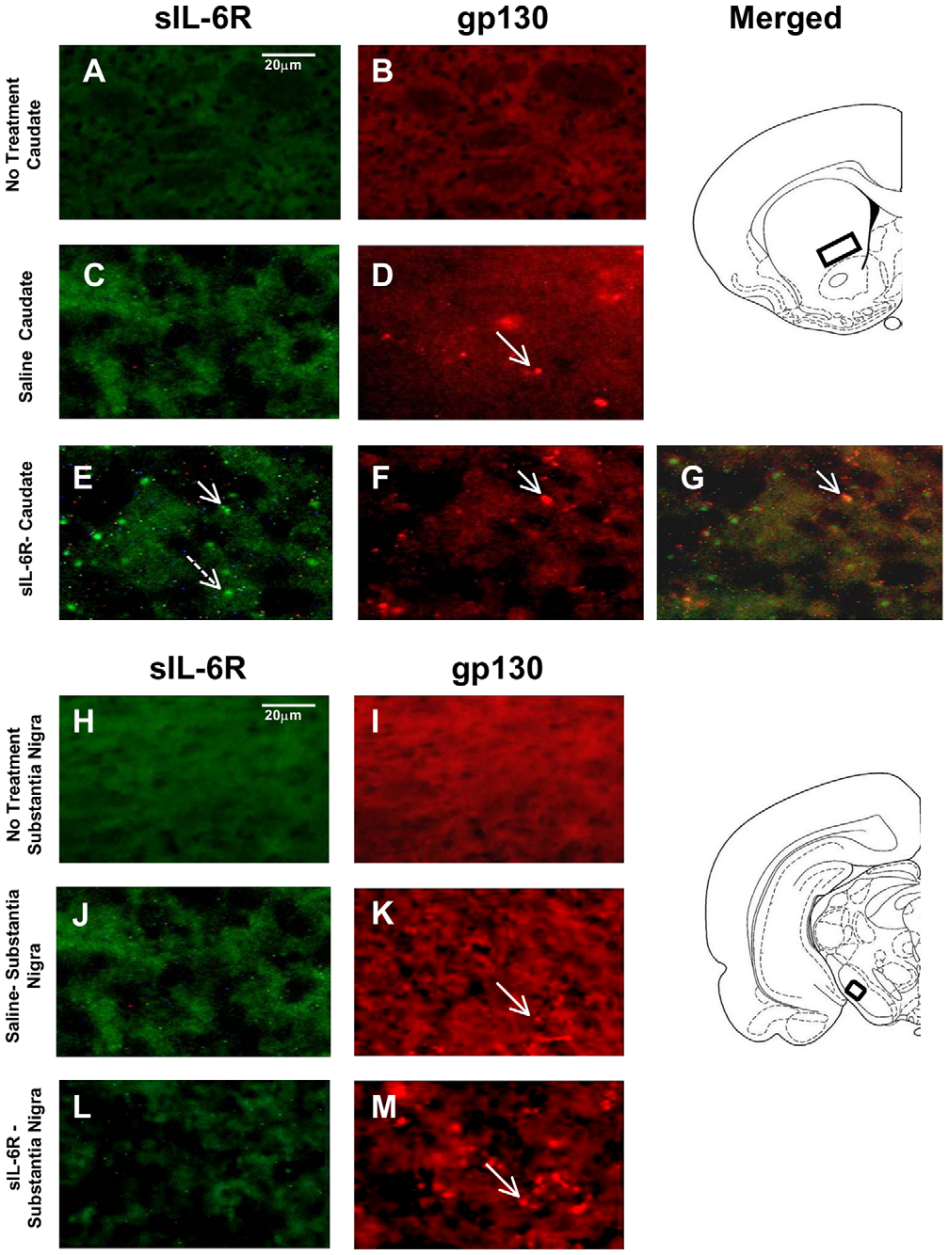
Fluorescent staining of Caudate Putamen and Substantia Nigra in sIL-6Rα treated mice. Photomicrographs of gp130 and sIL-6R labeling in caudate-putamen (CPu) and Substantia Nigra. (A) Absence of sIL-6R labeling in CPu of no treatment animal; (B) Absence of gp130 labeling in CPu of no treatment animal; (C) Absence of sIL-6R labeling in CPu of Saline treated animal; (D) Presence of gp130 labeling in CPu of Saline treated animal; (E) Presence of sIL-6R labeling in sIL-6R treated animal. (F) Presence of gp130 labeling in CPu of sIL-6R treated animal; (G) Co-localization of sIL-6R and gp130 in CPu of sIL-6R treated animal; (H) Absence of sIL-6R labeling in Substantia Nigra of no treatment animal; (I) Absence of gp130 labeling in Substantia Nigra of no treatment animal; (J) Absence of sIL-6R labeling in Substantia Nigra of Saline treated animal; (K) Presence of gp130 labeling in Substantia Nigra of Saline treated animal; (L) Absence of sIL-6R labeling in Substantia Nigra of sIL-6R treated animal; and (M) Presence of gp130 labeling in Substantia Nigra of sIL-6R treated animal. Arrows: illustration of both sIL-6R and gp130 labeling; Broken arrow: example of only single label of sIL-6R.

**Figure 5:**
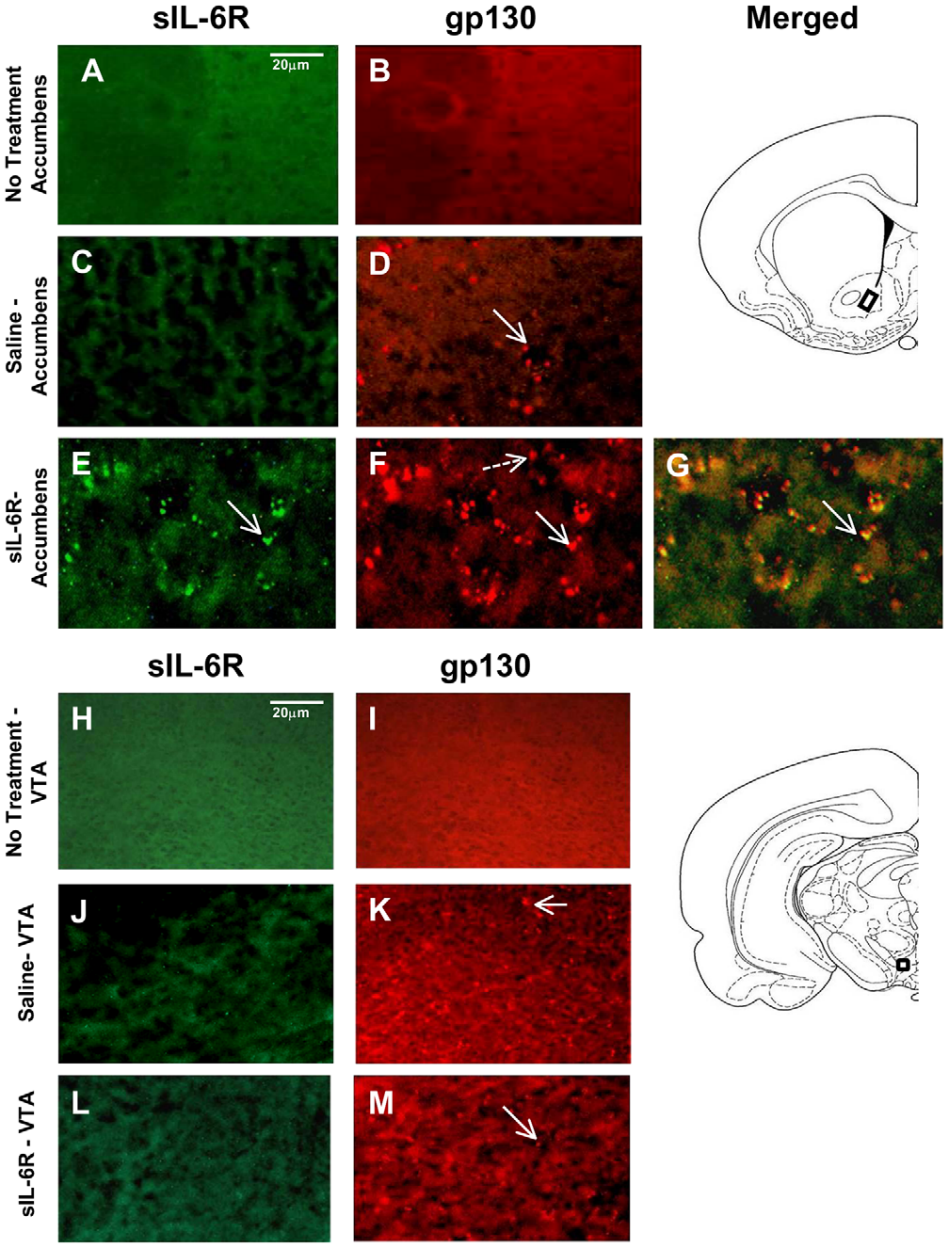
Fluorescent staining of Nucleus Accumbens and Ventral Tegmental Area in sIL-6Rα treated mice. Photomicrographs of gp130 and sIL-6R labeling in Nucleus Accumbens (NAc) and Ventral Tegmental Area (VTA). (**A**) Absence of sIL-6R labeling in NAc of no treatment animal; (**B**) Absence of gp130 labeling in NAc of no treatment animal; (**C**) Absence of sIL-6R labeling in NAc of Saline treated animal; (**D**) Presence of gp130 labeling in NAc of Saline treated animal; (**E**) Presence of sIL-6R labeling in NAc in sIL-6R treated animal. (**F**) Presence of gp130 labeling in NAc of sIL-6R treated animal; (**G**) Co-localization of sIL-6R and gp130 in NAc of sIL-6R treated animal; (**H**) Absence of sIL-6R labeling in VTA of no treatment animal; (**I**) Absence of gp130 labeling in VTA of no treatment animal; (**J**) Absence of sIL-6R labeling in VTA a of Saline treated animal; (**K**) Presence of gp130 labeling in VTA of Saline treated animal; (**L**) Absence of sIL-6R labeling in VTA of sIL-6R treated animal; and (**M**) Presence of gp130 labeling in VTA of sIL-6R treated animal. Arrows: illustration of both sIL-6R and gp130 labeling; Broken arrow: example of only single label of gp130.

**Figure 6:**
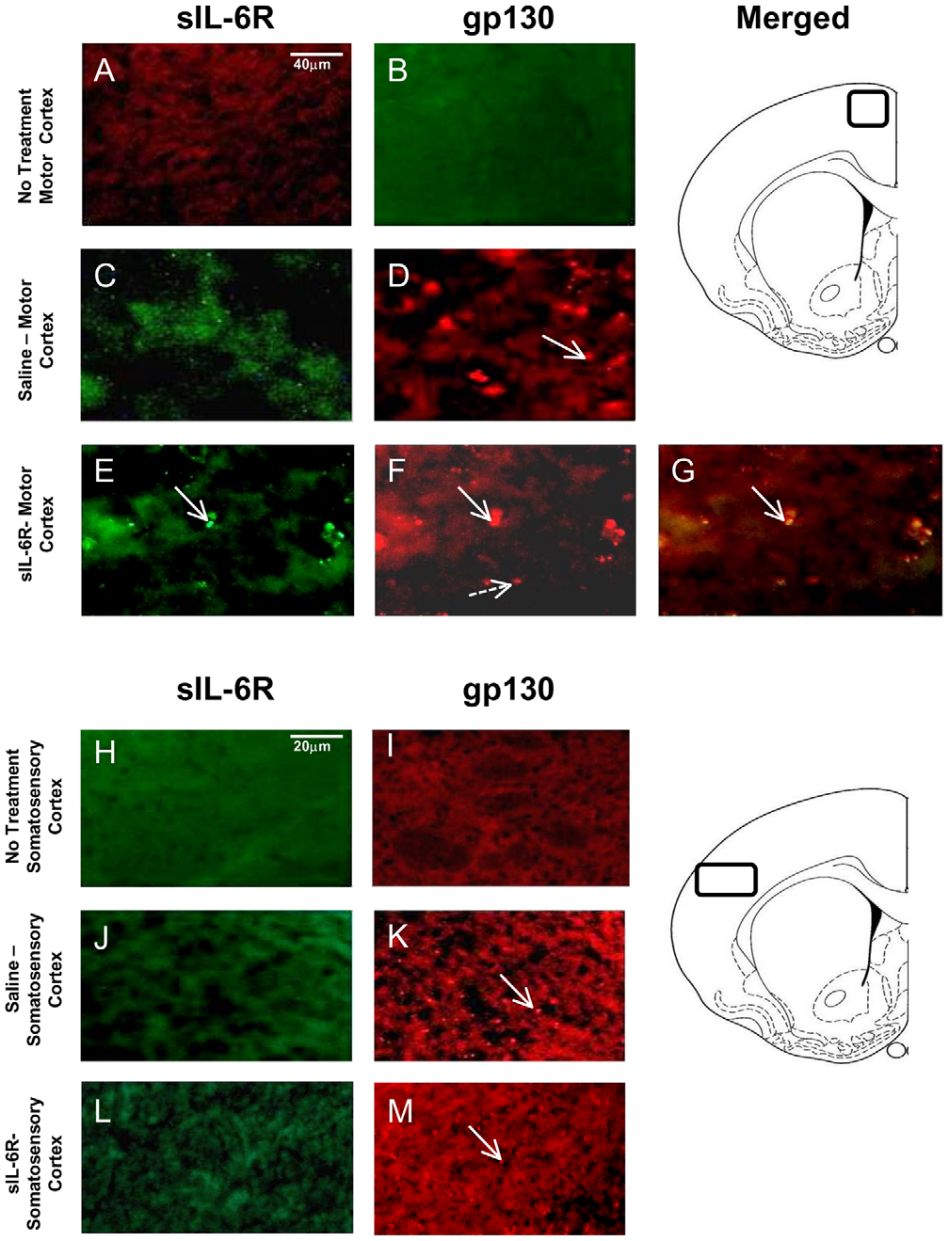
Fluorescent staining of Motor and Somatosensory Cortices in sIL-6Rα treated mice. Photomicrographs of gp130 and sIL-6R labeling in Motor Cortex (MCx) and Somatosensory Cortex (SSCx). (**A**) Absence of sIL-6R labeling in MCx of no treatment animal; (**B**) Absence of gp130 labeling in MCx of no treatment animal; (**C**) Absence of sIL-6R labeling in MCx of Saline treated animal**;** (**D**) Presence of gp130 labeling in MCx of Saline treated animal; (**E**) Presence of sIL-6R labeling in MCx in sIL-6R treated animal. (**F**) Presence of gp130 labeling in MCx of sIL-6R treated animal; (**G**) Co-localization of sIL-6R and gp130 in MCx of sIL-6R treated animal; (**H**) Absence of sIL-6R labeling in SSCx of no treatment animal; (**I**) Absence of gp130 labeling in SSCx of no treatment animal; (**J**) Absence of sIL-6R labeling in SSCx a of Saline treated animal; (**K**) Presence of gp130 labeling in SSCx of Saline treated animal; (**L**) Absence of sIL-6R labeling in SSCx of sIL-6R treated animal; and (**M**) Presence of gp130 labeling in SSCx of sIL-6R treated animal. Arrows: illustration of both sIL-6R and gp130 labeling; Broken arrow: example of only single label of gp130.

**Figure 7.Fluorescent:**
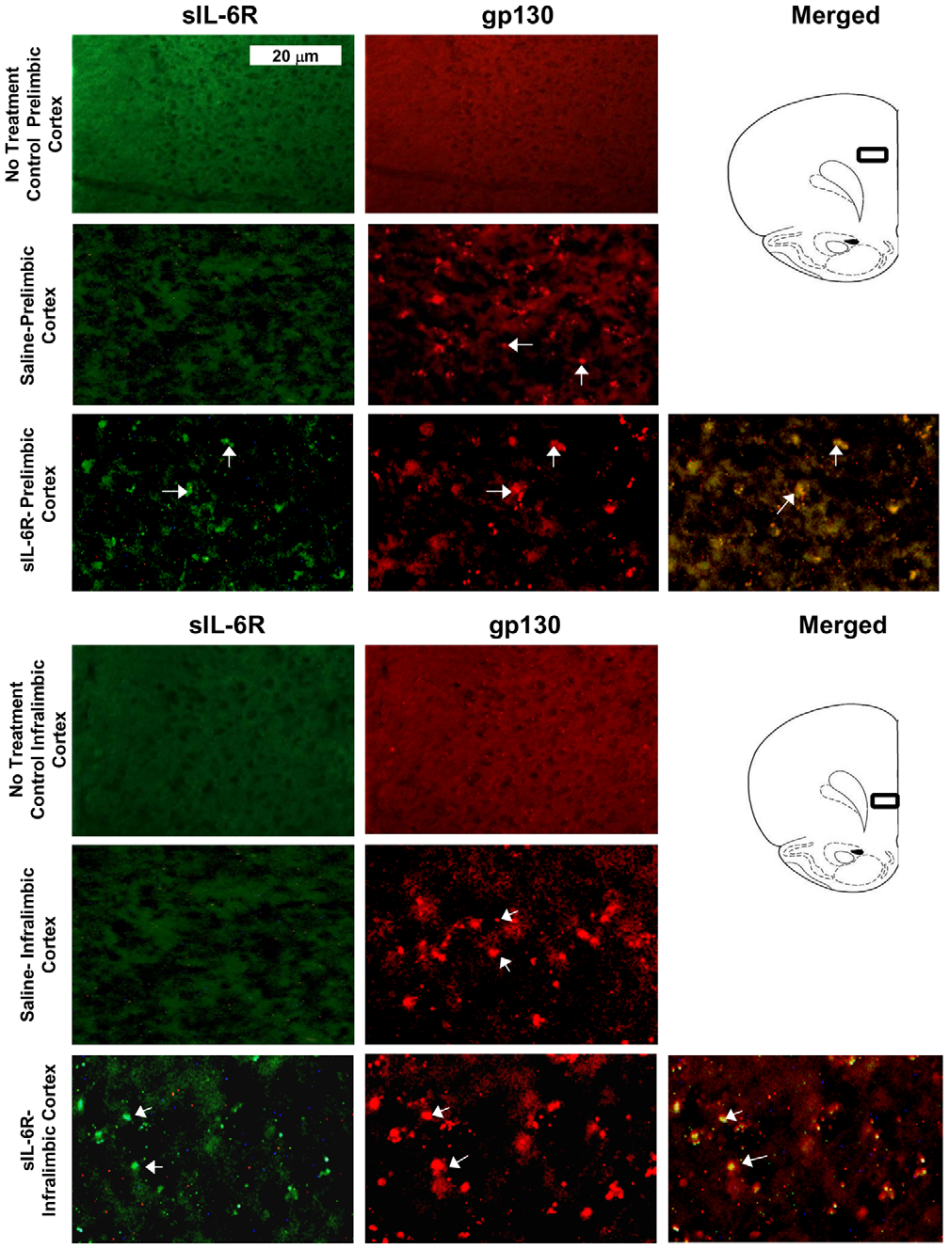
staining of Prelimbic and Infralimbic cortices in sIL-6Rα treated mice. Photomicrographs of gp130 and sIL-6R labeling in Prelimbic Cortex (PrLCx) and Infralimbic Cortex (ILCx). (**A**) Absence of sIL-6R labeling in PrLCx of no treatment animal; (**B**) Absence of gp130 labeling in PrLCx of no treatment animal; (**C**) Absence of sIL-6R labeling in PrLCx of Saline treated animal**;** (**D**) Presence of gp130 labeling in PrLCx of Saline treated animal; (**E**) Presence of sIL-6R labeling in PrLCx in sIL-6R treated animal. (**F**) Presence of gp130 labeling in PrLCx of sIL-6R treated animal; (**G**) Co-localization of sIL-6R and gp130 in PrLCx of sIL-6R treated animal; (**H**) Absence of sIL-6R labeling in ILCx of no treatment animal; (**I**) Absence of gp130 labeling in ILCx of no treatment animal; (**J**) Absence of sIL-6R labeling in ILCx a of Saline treated animal; (**K**) Presence of gp130 labeling in ILCx of Saline treated animal; (**L**) Presence of sIL-6R labeling in ILCx of sIL-6R treated animal; and (**M**) Presence of gp130 labeling in ILCx of sIL-6R treated animal. Arrows: illustration of both sIL-6R and gp130 labeling.

**Figure 8:**
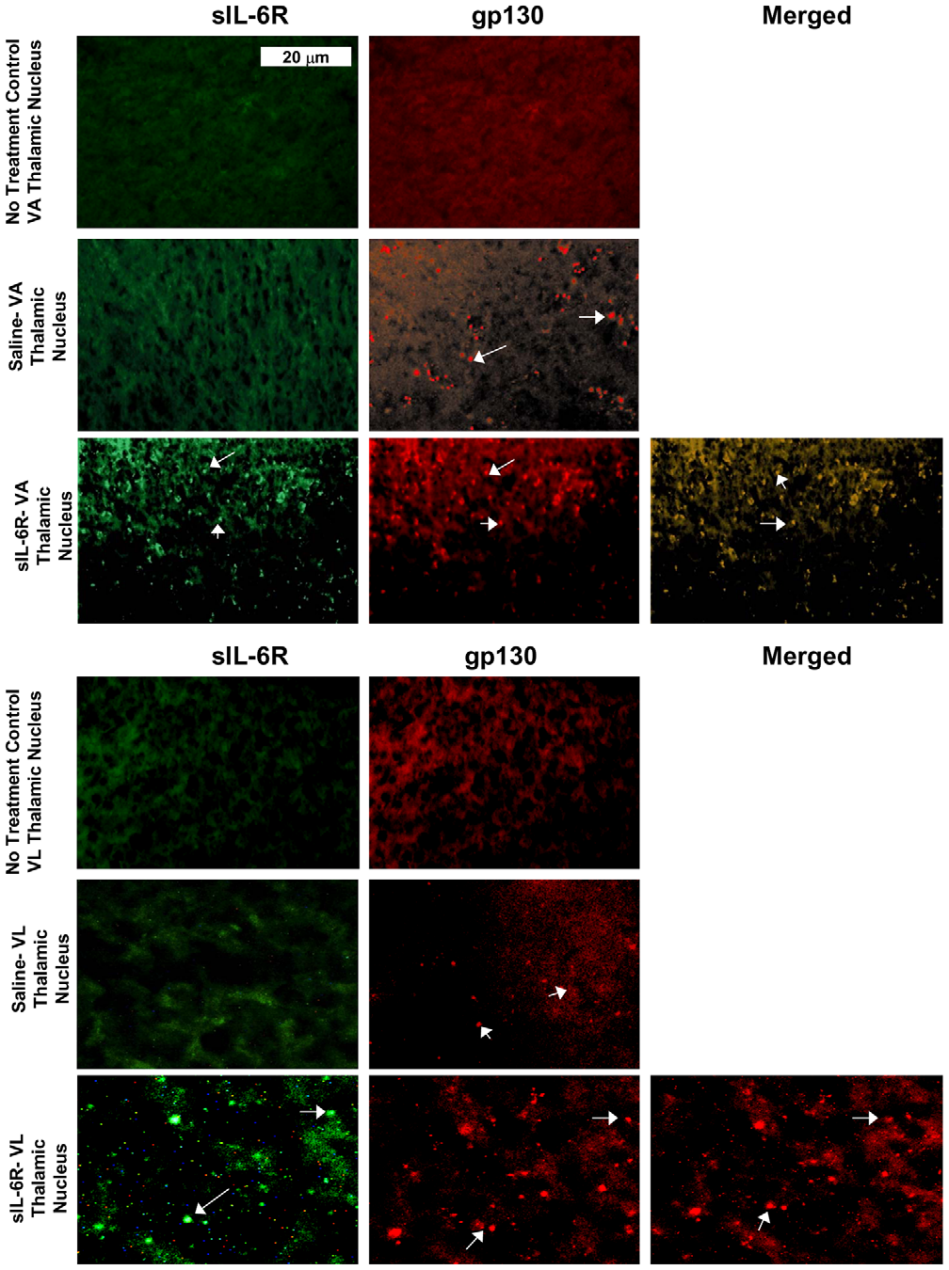
Fluorescent staining of Ventral Anterior and Ventral Lateral Thalamic Nuclei in sIL-6Rα treated mice. Photomicrographs of gp130 and sIL-6R labeling in Ventral Anterior Thalamic Nucleus (VATN) and Ventral Lateral Thalamic Nucleus (VLTN). (**A**) Absence of sIL-6R labeling in VATN of no treatment animal; (**B**) Absence of gp130 labeling in VATN of no treatment animal; (**C**) Absence of sIL-6R labeling in VATN of Saline treated animal**;** (**D**) Presence of gp130 labeling in VATN of Saline treated animal; (**E**) Presence of sIL-6R labeling in VATN in sIL-6R treated animal. (**F**) Presence of gp130 labeling in VATN of sIL-6R treated animal; (**G**) Co-localization of sIL-6R and gp130 in VATN of sIL-6R treated animal; (**H**) Absence of sIL-6R labeling in VLTN of no treatment animal; (**I**) Absence of gp130 labeling in VLTN of no treatment animal; (**J**) Absence of sIL-6R labeling in VLTN a of Saline treated animal; (**K**) Presence of gp130 labeling in VLTN of Saline treated animal; (**L**) Presence of sIL-6R labeling in VLTN of sIL-6R treated animal; and (**M**) Presence of gp130 labeling in ILCx of sIL-6R treated animal. (**N**) Co-localization of sIL-6R and gp130 in VLTN of sIL-6R treated animal. Arrows: illustration of both sIL-6R and gp130 labeling.

Given that levels of sIL-6R correlate with positive symptoms in schizophrenia and that sIL-6R can (regulate/modulate) IL-6 activity, it is noteworthy that administration of IL-6 to rodents induces behavioral changes, with single injections of IL-6 stimulating ambulatory exploration in Balb/c mice [18] and repeated injections increasing locomotion and emotional behaviors in rats [19]. In Balb/c mice, single peripheral injections of IL-6 stimulated exploratory motor activity [18] induced marked increases in dopamine and serotonin utilization in the medial prefrontal cortex, and increases in serotonin utilization in the dorsal hippocampus [20]. Peripheral IL-6 administration also increased brain tryptophan concentrations in mice [21]. Thus, IL-6 modulates the activity of neurotransmitters that are associated with the expression of positive symptoms in schizophrenia and targeted by antipsychotic drugs. In contrast to IL-6, there is no evidence that peripheral or central injections of sIL-6R alone can induce behavioral disturbances. In one study, intracerebroventricular injections of sIL-6R exaggerated IL-6-induced variations in locomotion in rats [22].

In view of the link between sIL-6R and positive symptoms in schizophrenics, coupled with the link between IL-6 and motor activity and neurotransmitters targeted by antipsychotic drugs, we sought to determine whether peripheral injections of sIL-6R stimulates exploratory activity and repetitive stereotypies. We also determined whether peripherally administered sIL-6R localized in brain regions associated with cortico-striatal-thalamo-cortical (CSTC) circuits, which are putative neuroanatomical substrates of disorders associated with repetitive motor stereotypies. In light of the relation between sIL-6R and gp130 in IL-6 trans-signaling, we also determined whether peripherally administered sIL-6R co-localize with gp130 in CSTC circuits.

## Materials and Methods

### Subjects

All methods and procedures were approved by Institutional Animal Care and Use Committee (IACUC) of University of Medicine and Dentistry of New Jersey (UMDNJ), Newark, New Jersey. A total of 98 adult male Balb/c mice were used in this study. The mice were purchased from Charles River Laboratories (Wilmington, MA). We used male Balb/c mice, based on our findings in these mice that peripheral IL-6 administration induces behavior activating effects [18] and modulate brain dopamine and serotonin activity [20]. The mice were housed in standard polypropylene ‘shoebox’ cages in groups of four until testing, maintained on a 12 hour light/12 hour dark light cycle (7 am –7 pm), and permitted free access to standard laboratory chow and water.

### Drugs

Recombinant human carrier-free soluble interleukin-6 receptor alpha (sIL-6Rα) (catalog #227-SR-025/CF) was purchased from R&D Systems (Minneapolis, MN). This form of sIL-6Rα is reactive to both human and mouse IL-6. Using human sIL-6Rα is advantageous as it permits the unambiguous identification of peripherally injected human sIL-6Rα in the mouse brain (Experiment 3). In these studies, anti-human IL-6Rα antibodies used to detect the human IL-6Rα did not detect the endogenous mouse IL-6R that may be present in the brain. The sIL-6Rα was injected subcutaneously (s.c.) (0–1 µg/mouse) in a volume of approximately 0.2 ml. The route of administration was based on our studies showing that monoclonal antibodies injected via the sc route gain entry into the brain and induce marked behavioral variations [23]–[27].

GBR 12909 (Tocris, Ballwin, MO), a highly selective dopamine transporter inhibitor with psychostimulant-like properties, was injected at a dose of 5 mg/kg (i.p.) in saline. We have determined that this dose induces a submaximal effect on motor activity and does not induce repetitive motor stereotypies; thus, one can determine whether combined treatment with sIL-6Rα and GBR 12909 has an additive effect on repetitive motor stereotypies.

### Behavioral Testing

In Experiment 1, mice (n = 11/group) were injected with various doses of sIL-6Rα (0–1 µg/mouse, s.c.) and immediately thereafter were individually placed into a test arena (TruScan Behavioral Monitoring System; Coulbourn Instruments, PA) for 2-hr in normal illumination. A series of behavioral measurements were recorded including locomotion, vertical activity, horizontal and vertical stereotypic movements, jumps, and turns. Locomotion was defined as the sum of all vectored coordinate changes in the floor plane, and included floor plane total movement distance less the stereotypic movement distance. Turns were defined as movements where the animal enters 4 radially-contiguous quadrants in ascending or descending order, without interruption. Vertical stereotypic movements were defined as the total number of coordinate changes ±1.499 beam spaces in the vertical plane and back to the original point that do not exceed 2 seconds apart. Three such movements must be made before a stereotypy episode starts. When it does, the qualifying 3 movements are included in the total number of moves.

The test sessions were also filmed with a VHS camera, and at a later date, an experienced rater blinded to the treatment groups scored the incidence and duration of stereotypic behaviors, which included rearing, sniffing, head bobbing, and intense grooming. We selected these stereotypies based on our studies examining the behavioral consequences of cytokines and other immune-derived elements [18], [27], and the use of such behaviors as animal analogs of repetitive stereotyped movements [28]. Head bobbing was defined as the time (seconds) engaged in repeated movement of the head in the vertical head plane. A minimum of 3-seconds of uninterrupted head bobbing was defined as one episode. The duration of such episodes was recorded with a stopwatch. Measurements were taken 20-, 40-, 60-, 80-, 100- and 120- minutes after injection in 2-minute epochs. Results are presented as totals (± SEM) for the entire session, and in 20-min intervals (± SEM).

In Experiment 2, we determined whether sIL-6R treatment influences GBR 12909-stimulated stereotypy. Mice received saline or sIL-6Rα (0.5 µg/mouse, s.c.; n = 6/group; dose based on the findings of Experiment 1) and were individually placed into a holding cage for 30-min, whereupon they received an injection of a submaximal dose of GBR 12909 (5 mg/kg, i.p). The 30-min interval between injections was chosen to ensure that behavioral effects of sIL-6R and GBR 12909 would overlap. Immediately thereafter, the mice were individually placed into a test arena (TruScan Behavioral Monitoring System; Coulbourn Instruments, PA) for 2-hr as described earlier. Behavior was measured as per Experiment 1. We additionally rated behavior using a stereotypy rating scale [29].

### Immunohistochemical analyses of peripherally administered sIL-6Rα in the brain

In Experiment 3, we determined whether peripherally injected human sIL-6Rα localize in brain regions associated with CSTC circuits, which are neuroanatomical substrates of the observed behavioral disturbances. Mice received single injections of human sIL-6Rα (0 or 0.5 µg/mouse, s.c.; n = 4/group) and immediately thereafter were individually placed into a test arena for 2 hours (as per Experiment 1). Immediately following the test session, the mice were deeply anesthetized with Na pentobarbital (60–80 mg/kg, IP) and transcardially perfused with 0.9% saline (pH 7.2) followed by 4% paraformaldehyde (pH 7.4). After perfusion, brains were removed from the skull and placed in 4% paraformaldehyde solution at 4°C for overnight. The brains were transferred to 30% sucrose solution and kept at 4°C until they sank to the bottom whereupon they were frozen in a cryostat (Leica CM1900) at −20°C for slow freezing. Sections were cut at 30 µm thickness and alternate sections were transferred to 24 well plates containing PBS and washed for 30 minutes. Brain sections were first blocked with 5% normal goat serum containing 0.3% Triton X-100 in PBS for 1 hour and then incubated with anti-human sIL-6Rα (Monoclonal Antibody) (1∶100 dilution) overnight at 4°C followed by incubation with goat anti-human IgG-FITC (green, 1∶200; Santa Cruz, CA) secondary antibody for 1 hour at room temperature. The slices were mounted on a slide, and viewed under an Olympus fluorescence microscope.

### Statistics

The results were analyzed with both one-way and two-way (antibody receptor over time) analyses of variance (ANOVA) followed by Newman-Keuls test for confirmation of post-hoc comparisons (α = 0.05).

## Results

### Effects of sIL-6R on novelty-induced motor activity

We determined whether a single peripheral injection of sIL-6Rα influence novelty-induced exploratory motor activity and repetitive stereotypies. sIL-6Rα administration induced significant increases in exploratory motor activity, including locomotion (F (3, 32)  = 3.76, p = 0.02; Fig. 1A), rearing (F (3, 29) = 3.74, p = 0.021) (Fig. 1C), and turning (F (3, 31)  = 6.42, p = 0.001; Fig. 1E). Newman-Keuls post-hoc comparisons (α = 0.05) revealed that compared to controls, the 0.5 µg dose of sIL-6Rα was most effective in inducing these effects. Of further importance, sIL-6Rα induced a pronounced increase in repetitive stereotypies, including head bobbing (F (3, 39)  = 3.80, p = 0.02; Fig. 2A), vertical stereotypy movements (F (3, 29)  = 3.38, p = 0.03; Fig. 2C) and vertical stereotypy episodes (F (3, 30)  = 4.32, p = 0.01; Fig. 2E).

Further examination of the data over 20-min intervals revealed time-dependent pattern of behavioral changes following sIL-6Rα injections. In particular, marked increases in novelty-induced exploratory motor behaviors were evident within 20–40 min of injection (Fig. 1B). Similar effects were observed for Rearing episodes (Fig. 1D) and Turning activity (Fig. 1F). While the magnitude of these effects declined thereafter, progressive increases in vertical stereotypies were expressed (i.e., 40–80 min post injection) and persisted for the remainder of the test session (Fig. 2B, 2D & 2F). The finding that the expression of repetitive stereotypies increased while exploratory motor behaviors declined implies selective effects on behavior.

Data recently published by our lab has shown that sIL-1R1 did not appreciably affect novelty-induced motor behaviors or repetitive stereotypies [30]. Thus, there is specificity with respect to the present behavioral effects induced by sIL-6R.

### Effects of sIL-6R pre-treatment on GBR 12909-stimulated behavioral changes

In Experiment 1, we discovered that sIL-6R induce exploratory motor activity and repetitive stereotypies; this is consistent with an IL-6-like agonist effect. In Experiment 2, we determined whether sIL-6R pre-treatment would affect streotypies induced by the dopamine uptake inhibitor GBR 12909.

Compared to mice receiving saline + GBR 12909, mice receiving sIL-6Rα + GBR 12909 showed a significant increase in the combined stereotypy score (F (1, 14)  = 17.04, p = 0.001; Fig. 3). Inspection of the data over 20-min intervals revealed that effects persisted throughout the 2-hr test session Analyses of specific stereotypies revealed that mice in the sIL-6Rα + GBR 12909 group did not show the range of stereotypies induced by sIL-6Rα alone; thus, the present effects do not simply reflect a carryover effect of sIL-6R pre-treatment. Since GBR 12909 is a highly selective dopamine transporter inhibitor, these findings also imply a link between sIL-6R and brain dopaminergic system.

### Localization of peripherally injected sIL-6Rs in brain regions associated with CSTC circuits

The findings of Experiments 1 and 2 imply that sIL-6Rα reaches brain regions associated with CSTC circuits, the putative neuroanatomical substrates of disorders associated with repetitive motor stereotypies. Previously, there was no evidence that peripherally injected soluble receptors localize in such regions. Accordingly, we determined whether peripherally injected human sIL-6R would localize in the cortex, striatum, and thalamus. As mentioned above, the anti-human IL-6Rα antibodies that were used to label IL-6Rα do not bind endogenous mouse IL-6R that may be present in the brain; thus, any labeled receptor represents exogenous sIL-6Rα.

In sIL-6Rα-treated mice, sIL-6Rα was found in the caudate-putamen (Fig. 4), nucleus accumbens (Fig. 5), motor and infralimbic cortices (Fig. 6 and 7), and ventral nuclei of the thalamus (Fig. 8). A striking finding was that sIL-6Rα co-localized with gp130 in these regions. Little or no label was detected in the ventral tegmental area, substantia nigra, or somatosensory cortex, although gp130 was expressed in these regions. These findings show a selectivity of sIL-6R labeling and perhaps suggest that sIL-6R does not target cell bodies within the mesolimbic and mesostriatal pathways.

Specificity of label is also demonstrated by the fact that human sIL-6R was not evident in saline controls. However, it is of interest that gp130 deposits were evident in saline-treated mice. Because saline controls were exposed to novelty stress for 2-hr immediately after injections, an intriguing possibility is that stressor exposure induced gp130 expression in the brain. To control for stress, we stained for gp130 in an additional group of controls that were not exposed to novelty stress. These control mice were deeply anesthetized immediately on arriving in the laboratory and transcardially perfused for subsequent IHC procedures. There was an absence of gp130 label in all of the brain regions examined in these unstressed controls (see Fig. 4, 5, 6, 7, and 8). These findings coupled with those in saline-treated mice exposed to novelty stress imply that stressor exposure induces gp130 expression in the brain.

## Discussion

The soluble interleukin-6 receptor (sIL-6R) is an approximately 50–60 kDa protein that is generated by shedding from the membrane or by differential mRNA splicing. sIL-6R is a normal constituent of body fluids that regulate cytokine activity in antagonistic and agonistic manners. sIL-6R may have antagonistic effects by binding IL-6 and thus preventing interactions between the cytokine and its membrane-bound counterpart [1]. It is important to note that the sIL-6R/IL-6 complex can act as an agonist for a cell that expresses gp130 even if that cell does not normally respond to IL-6 [8], thus greatly expanding its potential regulatory effects. However, relatively little is known about non-immune targets of sIL-6R.

Because the behavioral profile of sIL-6R is associated with activation of cortico-striato-thalamo-cortico (CSTC) circuits, we determined whether sIL-6R localizes in relevant cortico-striatal structures. Previous work has shown that antibodies are able to cross the BBB in amounts sufficient to affect behavior [23]–[26]. The mechanism used by antibodies, the extracellular pathway, to cross the BBB is available to molecules with pharmacokinetic profiles similar to antibodies, those of long residence time in blood and small volumes of distribution [31], We discovered that peripherally injected sIL-6R's localize within the caudate-putamen, nucleus accumbens, and motor cortex. Further specificity to these effects was evidenced by the absence of labeling: (1) in the omission controls, (2) no treatment controls, and (3) selective regions examined such as the substantia nigra, ventral tegmental area, somatosensory cortex and prepyriform cortex. It is of interest to note that the regions where localization of sIL-6R was most prominent included those areas which are normally associated with regulations of motor functions. Therefore, it is not surprising that sIL-6R has a capacity to regulate such motor functions as location and stereotypy which were examined in the present study. Of additional significance, sIL-6Rα co-localizes in these regions with gp130, a transmembrane protein through which sIL-6R modulate immune cell activity. This finding suggests perhaps, that a possible mechanism underlying sIL-6R modulation includes its interaction with gp130.

A curious finding observed in the present study was that the maximal behavioral response was observed following administration of a dose of 0.5 µg while a higher dose (1.0 µg) resulted in a minimal behavioral effect. While we do not have a clear-cut explanation that could account for the diminished effect at a higher dose, one possibility is that administration of the lower dose was associated with activation of endogenous IL-6 or its receptor which may not have been the case with the higher dose employed. Further analysis of this problem will be needed to resolve this issue.

Finally, it is also of interest to consider the distinction between stereotypic movements and those normally viewed as normal. Stereotypic movements characteristically lack function or purpose and can show repetition at high frequencies and for a long duration in contrast to motor responses viewed as normal which are typically purposeful in function. In particular, the “bobbing” response was of specific interest and was most unique in that it basically absent in normal animals. Our observations suggest the possibility that there is some unique feature with respect to the IL-6 receptor in Balb/c mice that induces and drives this form of motor response. One possibility is that there may exist in this species a more highly sensitive mechanism with regard to the activation of the IL-6 receptor. Alternatively, such sensitivity may relate to the signaling pathway for this receptor that would allow for the expression of the bobbing response. Future research along these lines could address this issue and be of heuristic value.

In summary, we showed for the first time that a peripheral soluble receptor (sIL-6Rα) implicated in psychiatric disorders could cross the BBB, co-localize with gp130 in CSTC circuits, and induce marked behavior activating effects characterized by an increased expression of repetitive stereotypies. Based on these findings, we suggest that sIL-6R shed from the cell surface of activated monocytes (and possibly other cells), can enter the brain to modulate activity in CSTC circuits associated with repetitive stereotyped movements, thus providing a mechanism by which schizophrenia and other disorders in which repetitive stereotypies are expressed could be promulgated.

To our knowledge, this is the first demonstration of a link between stress and brain gp130 expression. Based on this finding, we tentatively suggest that IL-6 trans-signaling plays a role in the brain's response to a stressful event. Future studies are needed to confirm such an effect, to find out potential mechanism underlying sIL-6R induced behavioral changes, and whether sIL-6R treatment further augments gp130 expression in the brain.
